# Posttraumatic Growth Inventory: Factor Structure in the Context of DSM-IV Traumatic Events

**DOI:** 10.5402/2012/937582

**Published:** 2011-12-08

**Authors:** Princess E. Osei-Bonsu, Terri L. Weaver, Susan V. Eisen, Jillon S. Vander Wal

**Affiliations:** ^1^Department of Psychology, Saint Louis University, 211 North Grand Boulevard, Shannon Hall, St. Louis, MO 63103, USA; ^2^Center for Health Quality, Outcomes & Economic Research (CHQOER), Edith Nourse Rogers Memorial Veterans Hospital, 200 Springs Road (152), Bedford, MA 01730, USA; ^3^Department of Health Policy & Management, Boston University School of Public Health, 715 Albany Street, Boston, MA 02118, USA

## Abstract

Studies examining the dimensionality of the Posttraumatic Growth Inventory (PTGI) have yielded varying results. To date, no study has investigated the measure's factor structure in the context of DSM-defined traumatic events. The present study examined the structure in an undergraduate student sample (*N* = 379) reporting DSM-IV Criterion-A potentially traumatic events. Confirmatory factor analysis (CFA) did not support the original five-factor structure. Follow-up exploratory factor analysis and CFA on random halves of the sample showed poor model fit for 1-, 3-, and 7-factor models. Results suggest that the PTGI factor structure is unclear amongst individuals with DSM-IV traumatic events, and continued use of the total score is most appropriate. Future directions including the utility of the PTGI factors are discussed.

## 1. Introduction

Evidence of perceived benefits following challenging life experiences has been documented since the 1980s. Tedeschi and Calhoun [1] coined the term *posttraumatic growth* to represent positive psychological transformation in the aftermath of a challenging life experience. They classified three themes of posttraumatic growth. One theme is a change in self-perception. Survivors have reported increased self-assurance, self-reliance, and competence in dealing with difficult situations. Another theme is a change in relationships with others. After a traumatic event, people reported that their experiences resulted in the rekindling of lost relationships and the acceptance of social support. A changed philosophy of life is the last theme. This theme includes an improved perspective on life, reappraisal of one's priorities, increased appreciation for one's existence, and stronger spiritual and religious beliefs. During scale development, these three domains were operationalized into 21 items that loaded onto five factors—Relating to Others (7 items), New Possibilities (5 items), Personal Strength (4 items), Spiritual Change (2 items), and Appreciation of Life (3 items). Together, the five factors make up the Posttraumatic Growth Inventory (PTGI), the most commonly used measure of posttraumatic growth.

Over the past 15 years, the PTGI has been used extensively. Researchers have assessed growth in various groups across the lifespan [2–4] and in survivors of high-magnitude stressors such as war, sexual assault, and life-threatening illnesses [5–9]. Some have translated the PTGI into different languages to assess growth in cross-cultural contexts such as refugees of the Bosnian war and Kosovo conflicts, survivors of the 2004 Madrid bombing, and Latina immigrants [10–13].

Despite widespread use of the measure, the dimensionality of the PTGI has been a topic of debate. The original five-factor solution was supported by the PTGI authors [14] as well as an independent research team [15]. However, other evidence suggests that the consideration of other factor structures is warranted. First, Linley et al. [15] cited two reasons for the need for further exploration of the PTGI structure: one, the original five-factor model did not show good fit across all indices; and two, a three-factor model representing the core themes of posttraumatic growth showed a moderate fit. Second, the PTGI contains two factors that have very few items—Spiritual Change and Appreciation of Life, which have only 2 and 3 items, respectively. A factor with fewer than three items can be considered weak and unstable [16]. Lastly, studies have consistently reported moderate-to-high correlations among the factors [1, 14, 17] raising the potential of overlapping constructs. For example, Taku et al. [14] noted poor discriminant validity between the New Possibilities and Personal Strength subscales. It is possible that the PTGI is best represented by fewer than five-factors.

In support of this possibility, there is empirical evidence for alternative factor structures including single- and three-factor models. In a sample of 136 refugees from the former Yugoslavia, Powell et al. [12] found a three-factor structure which corresponded to the following themes: Changes in Self/Positive Life Attitude, Philosophy of Life, and Relating to Others which map onto Tedeschi and Calhoun's original theoretical conceptualization of posttraumatic growth [1]. Other researchers have found that posttraumatic growth is best measured as a unitary construct in adult samples [3, 18], although neither used CFA. In addition to the three-factor model, Linley et al. [15] also found that a single higher-order construct with five first-order factors had an acceptable fit in a combined sample of British college students and adults endorsing a wide range of adverse experiences. Thus, the variation in the resulting factor structure may be due to differences in sample characteristics (e.g., college-age versus adult; U.S. versus foreign) and life experience (e.g., stressful versus traumatic events).

According to the Diagnostic and Statistical Manual of Mental Disorders—4th edition (DSM-IV; [19], traumatic events are stressors of “an extreme (i.e., life-threatening) nature” (Criterion A1) that elicit “intense fear, helplessness, or horror” (Criterion A2; [19]). Other feelings such as shame and guilt may also develop particularly as individuals engage in ruminative thought processes. In addition, extreme stressors can trigger the fight-flight reaction or freeze response [20]. In comparison, *stressful* experiences can be of any severity and can include events such as job loss, marital problems, and living in crime-ridden neighborhoods [19] that may not lead to intense sympathetic arousal. As the name suggests, *posttraumatic* growth can be activated after experiencing high-magnitude trauma-related events that go above and beyond *stressful* experiences [9, 21]. To date, no known study has examined the PTGI factor structure in a sample reporting DSM-IV-defined traumatic events. Given the widespread use of the PTGI with individuals reporting potentially traumatic events (e.g., sexual assault, war), assessing the validity of the use of the measure in such populations is warranted.

In the present investigation, we explored whether the original five-factor structure was replicable in a trauma-exposed undergraduate student sample—a sample similar to that with which the PTGI was developed, but restricted to those reporting DSM-IV-defined Criterion-A potentially traumatic events. The plan was to conduct a CFA on the five-factor solution. However, if the solution did not evidence an adequate fit, the plan was to employ a cross-validation paradigm whereby an exploratory factor analysis (EFA) was conducted using a random half of the study sample followed by a CFA in the remaining half.

## 2. Method

### 2.1. Participants

The total sample included 372 students enrolled from undergraduate psychology classes at a large Midwestern university. Students were eligible for the study if they were at least 18 years of age and experienced a Criterion-A potentially traumatic event as defined by the DSM-IV. One thousand forty-one students were recruited for this study. One student was under 18 years of age and was therefore excluded. Four hundred forty-nine did not endorse any of the 12 traumatic events. Another 219 were eliminated because they did not endorse helplessness or fear, or they selected “other” as their most bothersome event but did not provide a description of the event so we could not confirm that they were responding about a Criterion-A traumatic event. For the remaining 372 participants, the mean age was 19.5 years (SD = 1.9). The sample included 72% women and was 75% White. Other demographic information is presented in Table 1.

### 2.2. Procedure

This study was approved by the university Institutional Review Board. Participants were recruited for the study through announcements by psychology faculty and instructors who offered extra course credit for study participation. Students interested in participating either completed the measures on paper or logged onto a secure website specifically designed for research purposes. Informed consent was provided prior to the completion of measures.

### 2.3. Measures

#### 2.3.1. Demographic Questionnaire

Participants completed a brief self-report questionnaire to assess demographic information (age, gender, ethnicity, and year in school).

#### 2.3.2. Posttraumatic Diagnostic Scale (PDS)

The PDS is a four-part, 49-item self-report screening and diagnostic instrument typically used to assess the presence and severity of PTSD symptoms as outlined by DSM-IV [22]. The first two sections were used in this study to determine eligibility (i.e., endorsement of DSM-IV Criterion-A potentially traumatic events). These sections comprise a checklist of 12 potentially traumatic events (including a selection of the most bothersome event), and inquire about physical injury sustained and life endangerment associated with the most bothersome event (Criterion A1). They also assess subjective responses of helplessness and terror during that event (Criterion A2). Criterion-A was met if individuals endorsed the following: (1) at least one of the 12 events, (2) physical injury or life endangerment, and (3) helplessness or terror.

#### 2.3.3. Posttraumatic Growth Inventory (PTGI)

The PTGI is a 21-item, 6-point scale self-report measure (0 = I did not experience this change as a result of my crisis to 5 = I experienced this change to a very great degree as a result of my crisis; [1]). The measure was modified for the present study such that participants were asked to reselect their *most bothersome* potentially traumatic event prior to completing the PTGI items. They indicated the degree to which the statements were true of them as a result of their most bothersome traumatic event. The summation of all 21 items yielded a total growth score which can range from 0 to 105. Higher scores were indicative of greater growth. In the present study, internal consistency (Cronbach's *α*) of the total score was  .96 and item-total correlations ranged from  .59 to  .82.

### 2.4. Data Analysis

The original five-factor structure was tested in the complete study sample using LISREL version 8.8 [23]. With 21 PTGI items, this yields an indicator-to-factor ratio of approximately 4. According to Gagné and Hancock [24], using a sample between 200 and 400 participants (current *N* = 372) increases the likelihood model convergence. Because the data are ordinal, the CFA was performed on a polychoric correlation matrix with maximum likelihood (ML) estimation. And as recommended by Brown [25], three types of indices (absolute, parsimony correction, and incremental) were used to evaluate model fit.

Due to poor model fit, additional analyses were conducted in order to find a model with better fit. First, the sample was randomly divided into development and validation samples. Using SAS version 9.2 [26], EFA using ML extraction and promax rotation was performed on the development sample. ML extraction is appropriate for normally distributed constructs. Promax rotation is recommended when there is evidence of high correlation between factors [16], which is historically true for the PTGI factors [1, 14, 17]. Items with a minimum loading of 0.40 were retained and assigned to the factor on which they had the highest loading. While a minimum loading of 0.30 has been recommended, other factor analysis studies have used higher cutoffs to minimize cross-loading [3, 27]. Factor structures are stronger when cross-loadings are minimized [16]. CFAs using LISREL were conducted on the validation sample to assess the fit of the models obtained from the EFA based on the scree plot.

## 3. Results

### 3.1. Traumatic Events

Trauma experiences are presented in Table 2. The most frequently endorsed traumatic experiences were serious accident, life-threatening illness, natural disaster, nonsexual assault, other events (e.g., unexpected death of a loved one, witnessing a completed suicide), and sexual assault. Reporting of more than one event was common. About 55% of the sample reported two or more traumatic events. The mean number of traumatic events reported was 2.0 (SD = 1.2). The events most frequently reported as the *most bothersome* experiences were serious accident, life-threatening illness, and natural disaster. Despite a substantial number of traumatic events reported, overall, participants reported minimal trauma-related distress on the PDS (*M* = 8.0, SD = 9.5) as a result of their most bothersome traumatic experience.

### 3.2. Confirmatory Factor Analysis of Original Structure

We first performed a CFA of the original, correlated five-factor model in the total sample. The results showed a large chi-square statistic (*χ* ^2^). Due to the tendency for the *χ* ^2^ to be inflated due to large sample size, other indices were examined.  Table 3 presents three categories of fit indices for the original five-factor model—incremental (comparative fit index, CFI), absolute (goodness of fit index, GFI), and parsimony correction (root mean square error of approximation, RMSEA). While the CFI indicated excellent fit (above the  .95 cutoff), the GFI (below the  .90 cutoff) and RMSEA (above the  .08 cutoff) showed that the original five-factor model was a poor fit to the data. Examination of the modification indices showed that 17 out of the 20 items (item 16 was excluded as it did not meet the loading criteria) had a large modification index (>3.84), suggesting that they qualify for respecification [25].

### 3.3. Exploratory Factor Analysis—Development Sample

To find a factor model that best fit the data, an exploratory factor analysis was conducted on a random half (*n* = 176) of the sample. Eight factors with eigenvalues greater than one were extracted: New Path/Emotional Connectedness, Relating to Others, Personal Strength, Spiritual Change, Compassion and Change, Appreciation of Life, New Opportunities, and Positive Outlook. Five of the factors contained some or all of the items corresponding to the original PTGI factors.  Table 4 shows that 20 of the original 21 items had loadings greater than  .40 on one of the eight factors. Item 16 (I put more effort into my relationships) did not meet the cutoff on any of the factors. In addition, one factor had only one item and four factors had only two items. Interfactor correlations ranged from  .35 to  .61.

There is broad consensus in the literature that the eigenvalues >1 rule can lead to over-extraction and is one of the least accurate methods of extraction [28]. The scree test (examining the scree plot for natural breaks or bends) is a recommended alternative [16]. The scree test showed natural breaks at factors 2 and 4 which indicated that in addition to the 8-factor model, 1- and 3-factor solutions were possible. As a result, we proceeded with the following analyses: EFA specifying three factors using the development sample, and CFA to evaluate the fit of the 1-, 3-, and 8-factor models using the validation sample (*n* = 196).

The EFA with three specified factors yielded the following factors: Openness to Change, Self- and Other-Reliance, and Spiritual Change. Like the 8-factor solution, 20 out of 21 items loaded at least  .40 on the factors (Table 5). Item 2, I have a greater appreciation for the value of my own life, did not meet this threshold on any of the three factors. Notably, Spiritual Change was the only original factor reproduced in both exploratory analyses. 

### 3.4. Confirmatory Factor Analysis—Validation Sample

The CFA on the 8-factor model could not converge because the seventh factor, New Opportunities, contained only one item. As a result it was removed from the model. Fit indices for the 1-, 3-, and 7-factor models are presented in Table 3. All three models had a high *χ* ^2^ statistic, and their incremental fit index (CFI) showed acceptable fit. However indices for absolute fit (GFI) and parsimony correction (RMSEA) indicated poor model fit. Despite poor model fit for the 3- and 7-factor models, the item-total correlations for the new subscales in these models were all strong (>.50), and *α* coefficients showed good internal consistency (.80–.94) for each factor (Tables 6 and 7). Modification indices for the 3- and 7-factor models also suggested respecification for several items (9 and 19 items, respectively). 

## 4. Discussion

The goal of this study was to examine the factor structure of the PTGI in the context of DSM-IV Criterion-A potentially traumatic events within a sample of undergraduate students. Confirmatory analysis showed mixed support for these factors. The relative fit of this model (as indicated by the CFI) was acceptable; however, absolute fit indices (GFI and RMSEA) did not meet accepted fit criteria. Incremental fit indices, such as the CFI, often suggest adequate model fit [25]. As such, we conducted exploratory analysis to find the best model. It generated a new 8-factor solution—New Path/Emotional Connectedness, Relating to Others, Personal Strength, Spiritual Change, Compassion and Change, Appreciation of Life, New Opportunities (removed for confirmatory analysis), and Positive Outlook. While the relative fit was also acceptable, the absolute fit indices did not improve with the new factors. Exploration of other models yielded both a unitary and three-factor model as additional possibilities. However, fit indices did not improve with these models. Similar to Sheikh and Marotta [3], it appeared that the only stable factor in the current study was Spiritual Change, which was found in both the 3- and 7-factor models (as well as the original 5-factor model). 

Despite unacceptable fit statistics for all the confirmatory models tested, the internal consistency and item-total correlations for the all factors in each of the models were consistently high, suggesting that psychometrically they may not be distinct [29]. As noted earlier, there is substantial evidence that the original factors were highly intercorrelated. In addition, modification indices for the models tested in this study indicate multiple options for respecification of individual items. Decisions regarding scale revisions should be driven by the theoretical conceptualization of posttraumatic growth. For example, Tedeschi and Calhoun [1] conceptualized posttraumatic growth a construct with three underlying themes. If this conceptualization is maintained, themes should be operationally defined (e.g., what is meant by life philosophy?) and extraneous items removed. If there are indeed five subdomains of posttraumatic growth, the scale should be revised by adding a sufficient number of items to assess the underrepresented areas. If posttraumatic growth is best conceptualized as a single construct, there is much redundancy of measurement which would need elimination. 

There was one major methodological difference between the current study and the study by Taku et al. [14], which found support for the original five-factor structure. Taku et al. [14] treated the data as interval-level data. However, as a 6-point scale (0–5), the PTGI contains ordinal data. The use of polychoric correlations matrices in the current study treated the data as ordinal rather than interval. Linley et al. [15] used a matrix of polychoric correlations. However, they noted that the five-factor model did not demonstrate “close fit” according to the RMSEA and *χ* ^2^ statistic, which was also demonstrated in the current study. In fact, similar to the current study, Linley et al. found that none of the structures they explored (single higher-order, 3-factor, and 5-factor models) demonstrated good fit across all statistical indices. Taken together, it appears there is limited psychometric evidence that the specific factors are stable and provide any additional information beyond the total score. 

Other differences, between the present study and Tedeschi and Calhoun's [1] original study, are noteworthy. First, there was only a modest level of posttraumatic growth reported in this study. The total posttraumatic growth score for this study (*M* = 40.5) was considerably lower than that of Tedeschi and Calhoun's [1] study (*M* = 81.9). With the exception of restriction to DSM-IV events in this study, the two study samples were comparable in terms of age range, variety of experiences, and recency of event. In addition, the current sample was asked to report their posttraumatic growth as it relates to the event that was the *most bothersome*. The individuals in the original 1996 study were not given such directions and therefore may not have focused their report of posttraumatic growth on only one stressful experience. More than half of the current sample reported more than one traumatic event. Research in the area of PTSD assessment suggests that restricting respondents to one event can be problematic because they may have difficulty attributing their symptoms to a single event [30]. The same concept may apply to posttraumatic growth. It is possible that considering more than one event presents a greater opportunity for growth endorsement. 

Study limitations include the use of a convenience sample of undergraduate psychology students whose results may not generalize to other age groups. However, using a mixed trauma sample enhances the external validity of the study as 34.2% of men and 24.9% of women in the general population have experienced more than one trauma [31]. In addition, posttraumatic growth has been assessed in mixed trauma samples (e.g., [32]). Because there is no “gold standard” method for conducting factor analyses, we used recommended practices which may have impacted the results—variations in these practices may yield different results.

## 5. Conclusion

It remains unclear if posttraumatic growth following a traumatic experience, as measured by the PTGI, is best categorized as a multidimensional construct. Further research should rigorously explore the stability of individual factors and whether they capture unique aspects of posttraumatic growth. Studies should examine associations between these factors on mental health outcomes, functional status, and behavioral indicators. For example, are the factors related to PTSD and depression symptoms or quality of life? Are individuals who report changes in their ability to relate to others more socially active following their traumatic experience? Does spiritual change predict posttrauma religious and spiritual involvement? Demonstration of factor predictability provides evidence of discriminant validity of the factors and informs the predictive utility of the measure. In the meantime, preliminary evidence based on the current study suggests that continued use of the total score is recommended. Further refinement of the PTGI including possible deletion of redundant items or those that do not contribute to specific subscales, as well as addition of items to strengthen and/or better differentiate subscales might also be a fruitful avenue for additional research.

## Figures and Tables

**Table 1:** Sample characteristics.

	*n*	%
Age		
18	99	26.6
19	138	37.1
20	61	16.4
21	41	11.0
22 +	32	8.7
Missing	1	0.3

Gender		
Male	106	28.5
Female	266	71.5

Race		
White	279	75.0
Black	21	5.6
Asian	41	11.0
Other	17	4.6

Hispanic/Latino ethnicity		
Yes	14	3.8
No	358	96.2

Year in school		
Freshman	173	46.5
Sophomore	101	27.2
Junior	61	16.4
Senior	37	9.9

**Table 2:** Traumatic experiences.

	*n*	%
Lifetime traumatic experiences		
Serious accident	199	53.5
Life-threatening illness	142	38.2
Natural disaster	140	37.6
Nonsexual assault*	88	23.6
Other^†^	72	19.4
Sexual assault^‡^	64	17.1
Imprisonment	14	3.8
Military combat	11	3.0
Torture	5	1.5

Single versus multiple traumatic experiences		
1 event	166	44.6
≥2 events	206	55.4

PTGI event		
Serious accident	132	35.5
Life-threatening illness	96	25.8
Natural disaster	49	13.2
Nonsexual assault*	34	9.1
Sexual assault^‡^	31	8.3
Other^†^	17	4.6
Military combat	7	1.9
Imprisonment	3	0.8
Torture	3	0.8

Time since PTGI event		
<6 months	72	19.4
6 months to 3 years	127	34.1
3 to 5 years	76	20.4
>5 years	94	25.3
Unreported	3	0.8

*Includes nonsexual assault by a family member/someone known and nonsexual assault by a stranger.

^†^Includes unexpected death of a loved one, witnessing/learning of a suicide attempt or completed suicide, personal suicidality, and witnessing mass causalities.

^‡^Includes sexual assault by a family member/someone known, sexual assault by a stranger, and sexual contact under age 18 with someone 5 or more years older.

**Table 3:** Fit indices for models.

Model	*χ* ^2^	df	GFI	CFI	RMSEA	CI
Original 5-factor	1156.44	179	.77	.97	.12	.11–.13
7-factor	720.20	131	.72	.96	.15	.14–.16
3-factor	901.88	167	.68	.95	.15	.14–.16
1-factor	1115.62	189	.65	.94	.16	.15–.17

*Note.* GFI = goodness of fit index; CFI = comparative fit index; RMSEA = root mean square error of approximation; CI = 90% confidence interval.

**Table 4:** EFA factor loadings for the 8-factor solution.

PTGI item	NP/EC	RO	PS	SC	CC	AL	NP	PO
(1) I changed my priorities about what is important in life.	.40							
(3) I developed new interests.	.67							
(7) I established a new path for my life.	.69							
(8) I have a greater sense of closeness with others.	.60							
(9) I am more willing to express my emotions.	.64							
(6) I more clearly see that I can count on people in times of trouble.		.45						
(20) I learned a great deal about how wonderful people are.		.80						
(21) I better accept needing others.		.58						
(4) I have a greater feeling of self-reliance.			.42					
(10) I know better that I can handle difficulties.			.61					
(19) I discovered that I'm stronger than I thought I was.			.89					
(5) I have a better understanding of spiritual matters.				.95				
(18) I have a stronger religious faith.				.65				
(15) I have more compassion for others.					.44			
(17) I am more likely to try to change things which need changing.					.89			
(2) I have a greater appreciation for the value of my own life.						.57		
(13) I can better appreciate each day.						.61		
(14) New opportunities are available which wouldn't have been otherwise.*							.89	
(11) I am able to do better things with my life.								.42
(12) I am better able to accept the way things work out.								.76

*Cronbach's *α**	*.86*	*.80*	*.86*	*.87*	*.83*	*.85*	*—*	*.86*

*Note.* NP/EC = new path/emotional connectedness; RO = relating to others; PS = personal strength; SC = spiritual change; CC = compassion and change; AL = appreciation of life; NP = new possibilities; PO = positive outlook.

*Removed for confirmatory factor analysis.

**Table 5:** EFA factor loadings for the 3-factor solution.

PTGI item	OC	SOR	SC
(1) I changed my priorities about what is important in life.	.55		
(3) I developed new interests.	.81		
(4) I have a greater feeling of self-reliance.	.50		
(7) I established a new path for my life.	.94		
(8) I have a greater sense of closeness with others.	.44		
(9) I am more willing to express my emotions.	.51		
(11) I am able to do better things with my life.	.46		
(12) I am better able to accept the way things work out.	.46		
(14) New opportunities are available which wouldn't have been otherwise.	.47		
(15) I have more compassion for others.	.43		
(17) I am more likely to try to change things which need changing.	.56		
(6) I more clearly see that I can count on people in times of trouble.		.41	
(10) I know better that I can handle difficulties.		.67	
(13) I can better appreciate each day.		.58	
(16) I put more effort into my relationships.		.44	
(19) I discovered that I'm stronger than I thought I was.		.80	
(20) I learned a great deal about how wonderful people are.		.81	
(21) I better accept needing others.		.76	
(5) I have a better understanding of spiritual matters.			.77
(18) I have a stronger religious faith.			.94

*Cronbach's *α**	*.94*	*.90*	*.87*

*Note.* OC = openness to change; SOR = self- and other-reliance; SC = spiritual change.

**Table 6:** Item-total correlations and internal consistency for 3-factor structure.

Factor/item	Corrected item-total correlation	Cronbach's *α*
*Openness to change*		*.94*

(1) I changed my priorities about what is important in life.	.68	
(3) I developed new interests.	.68	
(4) I have a greater feeling of self-reliance.	.73	
(7) I established a new path for my life.	.76	
(8) I have a greater sense of closeness with others.	.69	
(9) I am more willing to express my emotions.	.72	
(11) I am able to do better things with my life.	.88	
(12) I am better able to accept the way things work out.	.78	
(14) New opportunities are available which wouldn't have been otherwise.	.59	
(15) I have more compassion for others.	.71	
(17) I am more likely to try to change things which need changing.	.85	

*Self- and other-reliance*		*.90*

(6) I more clearly see that I can count on people in times of trouble.	.54	
(10) I know better that I can handle difficulties.	.71	
(13) I can better appreciate each day.	.68	
(16) I put more effort into my relationships.	.74	
(19) I discovered that I'm stronger than I thought I was.	.72	
(20) I learned a great deal about how wonderful people are.	.74	
(21) I better accept needing others.	.75	

*Spiritual change*		*.87*

(5) I have a better understanding of spiritual matters.	.77	
(18) I have a stronger religious faith.	.77	

**Table 7:** Item-total correlations and internal consistency for 7-factor structure.

Factor/item	Corrected item-total correlation	Cronbach's *α*
*New path/emotional connectedness*		*.86*

(1) I changed my priorities about what is important in life.	.64	
(3) I developed new interests.	.66	
(7) I established a new path for my life.	.72	
(8) I have a greater sense of closeness with others.	.69	
(9) I am more willing to express my emotions.	.72	

*Relating to others*		*.80*

(6) I more clearly see that I can count on people in times of trouble.	.58	
(20) I learned a great deal about how wonderful people are.	.70	
(21) I better accept needing others.	.65	

*Personal strength*		*.86*

(4) I have a greater feeling of self-reliance.	.70	
(10) I know better that I can handle difficulties.	.75	
(19) I discovered that I'm stronger than I thought I was.	.75	

*Spiritual change*		*.87*

(5) I have a better understanding of spiritual matters.	.77	
(18) I have a stronger religious faith.	.77	

*Compassion and change*		*.83*

(15) I have more compassion for others.	.71	
(17) I am more likely to try to change things which need changing.	.71	

*Appreciation of life*		*.85*

(2) I have a greater appreciation for the value of my own life.	.74	
(13) I can better appreciate each day.	.74	

*Positive outlook*		*.86*

(11) I am able to do better things with my life.	.76	
(12) I am better able to accept the way things work out.	.76	
